# Urgent da Vinci-Assisted Laparoscopic Repair of an Incarcerated Adult Bochdalek Hernia Presenting With Large-Bowel Obstruction: A Case Report and Literature Review

**DOI:** 10.7759/cureus.114788

**Published:** 2026-08-19

**Authors:** Orlando Martinez Rosales, Ricardo Mohammed

**Affiliations:** 1 General Surgery, North Okaloosa Medical Center, Crestview, USA; 2 Bariatric and General Surgery, North Okaloosa Medical Center, Crestview, USA

**Keywords:** adult bochdalek hernia, congenital diaphragmatic hernia (cdh), da vinci robot, incarceration, large-bowel obstruction, robotic-assisted repair

## Abstract

Bochdalek hernias (BHs) are a rare form of diaphragmatic hernia in adults. They are typically diagnosed in infancy or childhood; however, in rare cases, they may be discovered in completely asymptomatic adults. Because this condition is uncommon, it can be mistaken for other conditions and misdiagnosed, exposing patients to unnecessary treatments. We present the case of a 33-year-old man with a past medical history of hypertension who presented to the ED with a four-day history of severe abdominal pain, nausea, and vomiting. CT of the abdomen and pelvis with contrast showed findings consistent with a mechanical large-bowel obstruction secondary to an incarcerated BH containing the splenic flexure of the colon and omental fat. The patient was successfully treated using a da Vinci-assisted laparoscopic approach with mesh repair. At the three-month follow-up, the patient remained asymptomatic, without clinical signs of recurrence. In adults, a BH is an uncommon form of diaphragmatic hernia. As many cases are asymptomatic, a high index of suspicion is needed because diagnosis can be challenging. Modern surgical techniques can improve patient recovery and result in shorter hospital stays, with minimal morbidity and mortality. In this case report, we demonstrate that a robot-assisted laparoscopic approach was a safe and effective means of repairing this hernia and provide an overview of the current literature.

## Introduction

Congenital diaphragmatic hernias (CDHs) are rare conditions, with a reported incidence of approximately one in 5,000 live births, and include several subtypes. Bochdalek hernias (BHs) are the most common subtype and arise from a developmental failure of the posterolateral pleuroperitoneal canal to close, allowing abdominal contents to herniate into the thorax and compress the developing lung parenchyma [1-6].

Regarding the embryology of BHs, this is said to occur during the fifth week of gestation, when the pleuroperitoneal membranes of the diaphragm form laterally, with the right side forming before the left side, toward the diaphragm’s septum transversum (ultimately called the central tendon). When there is a defect in the formation of the pleuroperitoneal membranes of the diaphragm, this creates a thinned segment or opening in the diaphragm and can ultimately allow abdominal contents to herniate into the thoracic cavity. This can lead to compression of the lungs, which can cause respiratory complications such as lung hypoplasia and pulmonary hypertension. This can result in respiratory distress, as well as bowel strangulation and bowel ischemia [1,2,5].

BHs are commonly diagnosed during the early postnatal period or in infancy [1-6]. In adults, however, they are often discovered incidentally on imaging or diagnosed after patients present with pulmonary or gastrointestinal symptoms. Estimates of their prevalence in adults vary considerably according to the population and imaging method studied [1-3]. The clinical presentation can vary, may include both abdominal and chest symptoms, and is influenced by the presence, severity, and acuteness of the herniation. Adult BHs are more commonly associated with gastrointestinal symptoms than with pulmonary symptoms. However, some cases may be asymptomatic and detected incidentally, particularly when the defect and volume of herniated contents are small [1-6].

In adults, incarceration of intrathoracic abdominal structures can lead to bowel strangulation and ischemia, requiring urgent surgical intervention [1,3,7]. The variable and often nonspecific presentation can make the diagnosis challenging and may delay appropriate treatment. Surgical repair remains the primary treatment strategy for symptomatic CDHs in adults. Minimally invasive techniques have become increasingly prevalent in surgery and are now being applied to diaphragmatic hernia repair in adults, including laparoscopy, thoracoscopy, and robot-assisted surgery [1,3,6,8-15].

Robot-assisted surgery has rapidly emerged and is now used for diaphragmatic hernia repair. With the maturation of robotic platforms, transabdominal and transthoracic robotic approaches appear to be effective treatment options, offering surgeons improved visualization, precision, and dexterity within a limited space; however, the published evidence remains limited and consists mainly of case reports and small case series [15].

We report a case of da Vinci-assisted laparoscopic repair of an incarcerated BH in an adult. We also review the current literature to highlight the clinical presentation, diagnostic considerations, and potential role of robot-assisted repair in this rare condition.

## Case presentation

**Figure 1:**
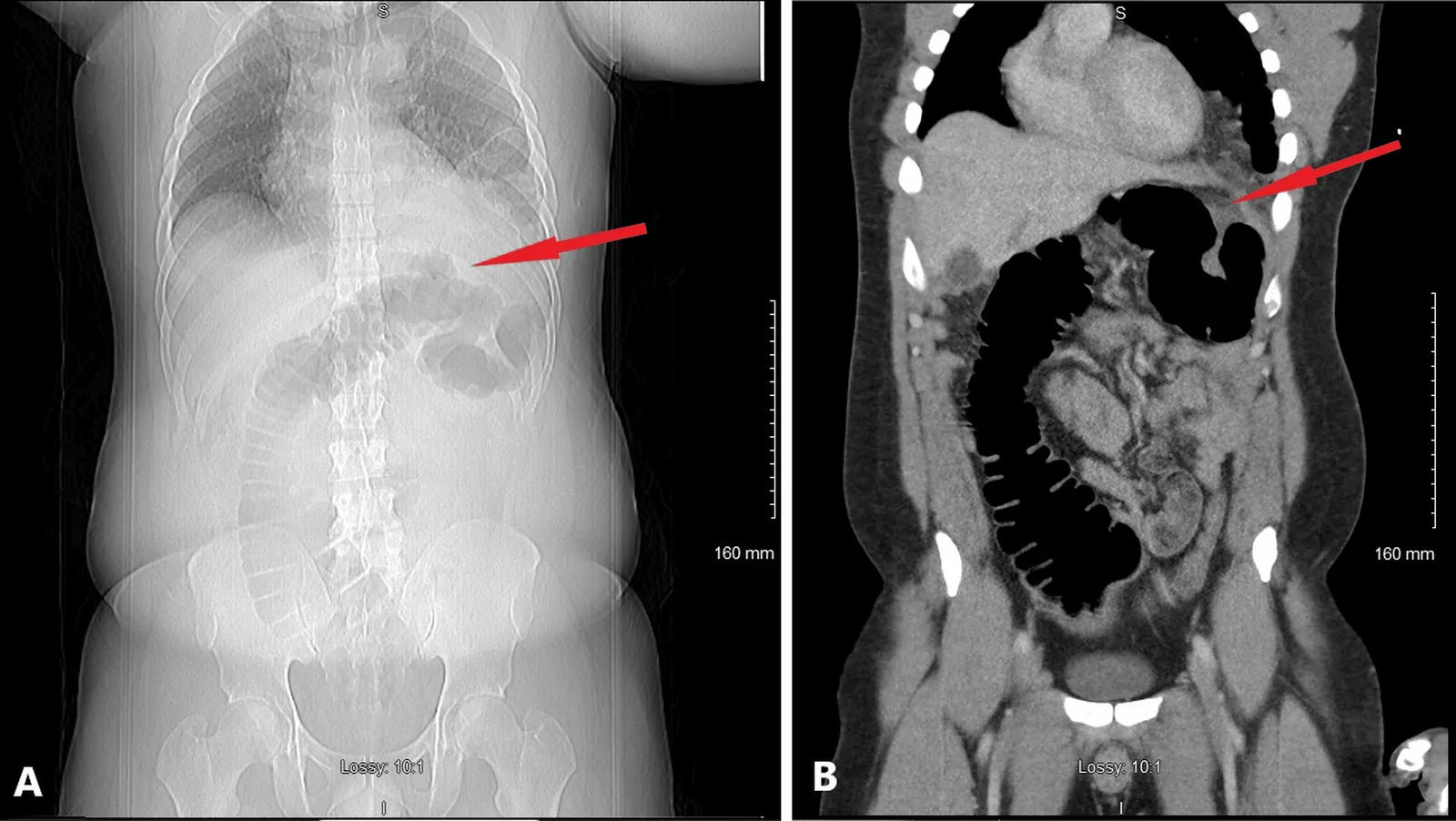
Preoperative noncontrast computed tomography. (A) Computed tomography scout image demonstrates gas-containing bowel loops projecting over the left lower hemithorax (red arrow), suggesting transdiaphragmatic herniation. (B) Coronal reconstruction demonstrates a left-sided posterolateral diaphragmatic (Bochdalek) defect (red arrow), with herniation of intra-abdominal contents, including bowel loops and omental tissue, into the left hemithorax.

**Figure 2:**
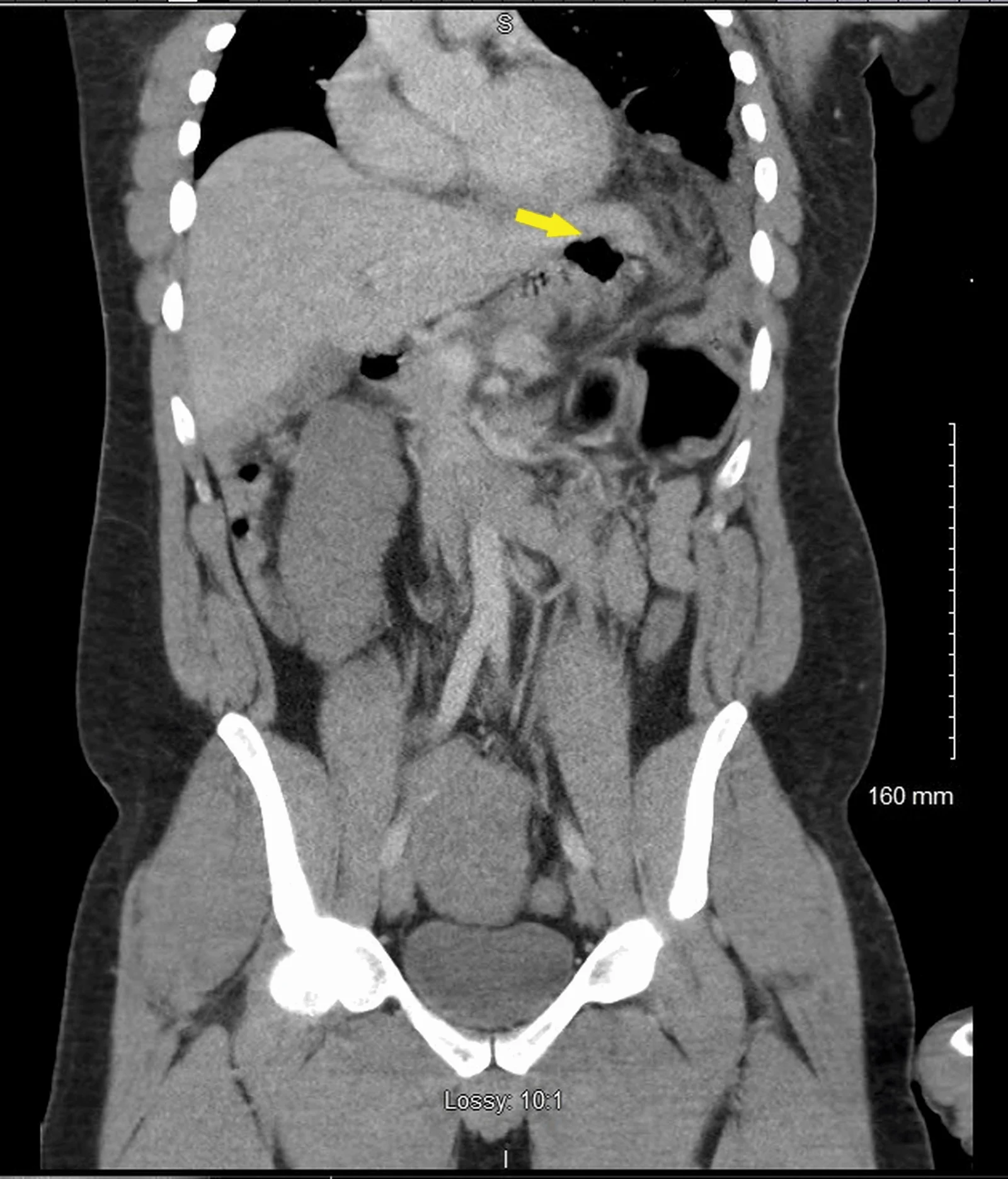
Preoperative contrast-enhanced computed tomography. The image demonstrates a left posterolateral diaphragmatic defect (yellow arrow) through which the splenic flexure and omental fat have herniated. The transition point and adjacent inflammatory changes are consistent with mechanical obstruction and raise concern for incarceration.

A 33-year-old man presented to the ED with a four-day history of severe abdominal pain, nausea, and vomiting. He appeared acutely ill and was moderately dehydrated. He had not had a bowel movement in the past four days. His medical history included hypertension and hyperlipidemia, and his medications included amlodipine, aspirin, and atorvastatin. He had no history of abdominal or thoracic surgery, trauma, or congenital anomalies. On presentation, his temperature was 36.7°C (98.2°F), blood pressure was 163/109 mm Hg, heart rate was 120 beats/min, and respiratory rate was 20 breaths/min. On physical examination, the patient was lying in bed and appeared somnolent, with dry mucous membranes. His abdomen was soft and nondistended, with diffuse tenderness and hypoactive bowel sounds. The remainder of the physical examination was unremarkable. CT of the abdomen and pelvis, performed before and after IV contrast administration, demonstrated a left-sided diaphragmatic hernia containing the splenic flexure of the colon and a portion of the omentum, resulting in mechanical large-bowel obstruction at the level of the hernia. Inflammatory changes in the herniated omental fat and mural thickening of the affected colonic segment raised concern for incarceration (Figures 1-2).

Initial laboratory findings are summarized in Tables 1-2. The complete blood count showed mild leukocytosis without anemia, with relative neutrophilia and lymphopenia. Serum chemistry testing demonstrated mild hyponatremia, hypochloremia, and mild hyperglycemia. Renal function, hepatic biochemical tests, total bilirubin, and serum lipase were within normal limits.

**Table 1:** Initial hematologic findings.

Parameter	Patient value	Adult reference range/threshold
WBC count	11.2 × 10³/µL	4.5-11.0 × 10³/µL
RBC count	5.46 × 10⁶/µL	4.50-5.90 × 10⁶/µL
Hemoglobin	14.5 g/dL	13.5-17.5 g/dL
Hematocrit	43.80%	41.0-53.0%
Mean corpuscular volume	80.1 fL	80.0-100.0 fL
Mean corpuscular hemoglobin	26.6 pg	26.0-34.0 pg
Mean corpuscular hemoglobin concentration	33.2 g/dL	32.0-36.0 g/dL
Platelet count	393 × 10³/µL	150-450 × 10³/µL
Mean platelet volume	6.2 fL	7.5-11.5 fL
Red cell distribution width-CV	13.50%	11.5-14.5%
Red cell distribution width-SD	38.5 fL	39.0-46.0 fL
Neutrophils	82.40%	40.0-70.0%
Absolute neutrophil count	9.2 × 10³/µL	1.5-7.5 × 10³/µL
Lymphocytes	9.30%	20.0-40.0%
Absolute lymphocyte count	1.0 × 10³/µL	1.0-4.0 × 10³/µL
Monocytes	7.80%	2.0-8.0%
Absolute monocyte count	0.7 × 10³/µL	0.2-0.8 × 10³/µL
Eosinophils	0.10%	0.0-5.0%
Absolute eosinophil count	0.0 × 10³/µL	0.0-0.5 × 10³/µL
Basophils	0.40%	0.0-1.0%
Absolute basophil count	0.0 × 10³/µL	0.0-0.2 × 10³/µL
Monocyte distribution width	15.4	≤20.0

**Table 2:** Initial chemistry findings.

Parameter	Patient value	Adult reference range
Glucose	121 mg/dL	70-99 mg/dL
Blood urea nitrogen	9 mg/dL	7-20 mg/dL
Creatinine	1.20 mg/dL	0.70-1.30 mg/dL
Estimated glomerular filtration rate	>60 mL/min/1.73 m²	≥60 mL/min/1.73 m²
Sodium	134 mmol/L	135-145 mmol/L
Potassium	3.6 mmol/L	3.5-5.0 mmol/L
Chloride	97 mmol/L	98-107 mmol/L
Carbon dioxide	29 mmol/L	22-29 mmol/L
Anion gap	12 mmol/L	8-12 mmol/L
Calcium	9.4 mg/dL	8.6-10.2 mg/dL
Total protein	7.9 g/dL	6.0-8.3 g/dL
Albumin	4.0 g/dL	3.5-5.0 g/dL
Alkaline phosphatase	81 U/L	44-147 U/L
Aspartate aminotransferase	13 U/L	10-40 U/L
Alanine aminotransferase	17 U/L	7-56 U/L
Total bilirubin	0.7 mg/dL	0.2-1.2 mg/dL
Serum lipase	20 U/L	13-60 U/L

Because of the mechanical large-bowel obstruction and concern for incarceration, the patient underwent urgent operative exploration following resuscitation and informed consent. Preoperative antibiotic prophylaxis with cefazolin was administered. Sequential compression devices were applied for mechanical thromboprophylaxis. The patient was brought to the operating room and prepared and draped in the usual sterile manner. Under general endotracheal anesthesia, the patient was placed in the supine position for a robot-assisted transabdominal approach using the da Vinci Xi Surgical System (Intuitive Surgical, Sunnyvale, CA). Pneumoperitoneum was established using a Veress needle, ports were placed under direct visualization, and the robotic platform was docked.

**Figure 3:**
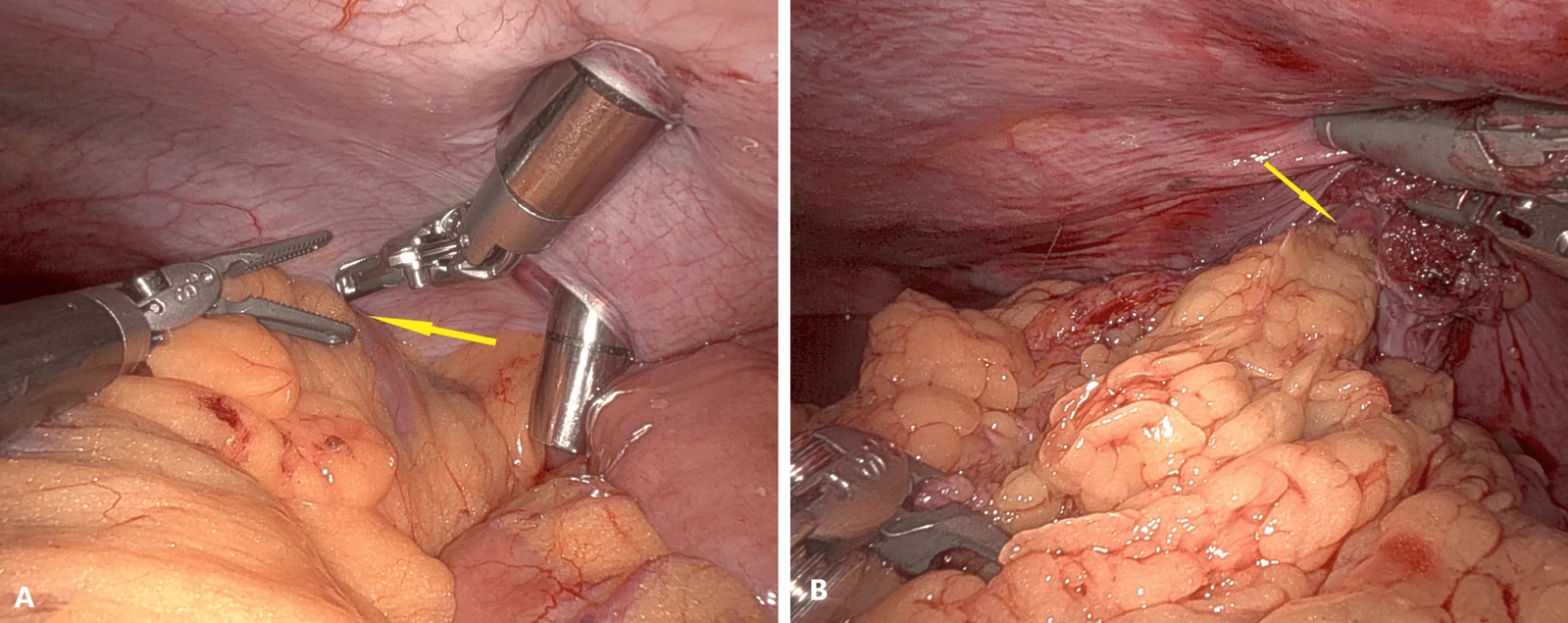
Intraoperative view. Intraoperative view of the left-sided Bochdalek hernia before reduction, showing incarceration of the splenic flexure of the colon and a portion of the omentum within the posterolateral diaphragmatic defect (yellow arrow).

**Figure 4:**
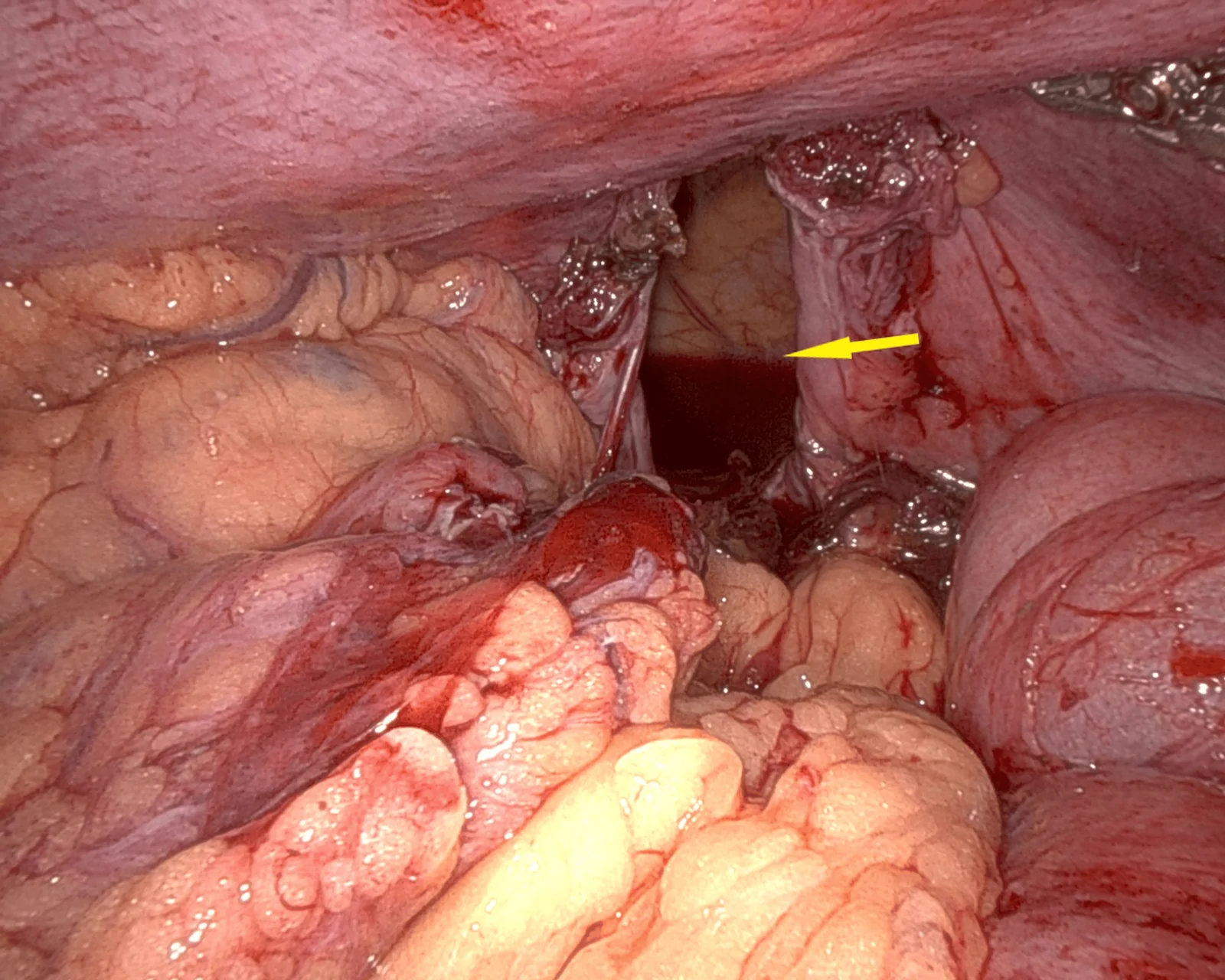
Intraoperative view of the hernia defect following reduction of the herniated contents. A large diaphragmatic defect is visible in the posterior aspect of the left hemidiaphragm, with clear visualization of the thoracic cavity through the defect (yellow arrow).

**Figure 5:**
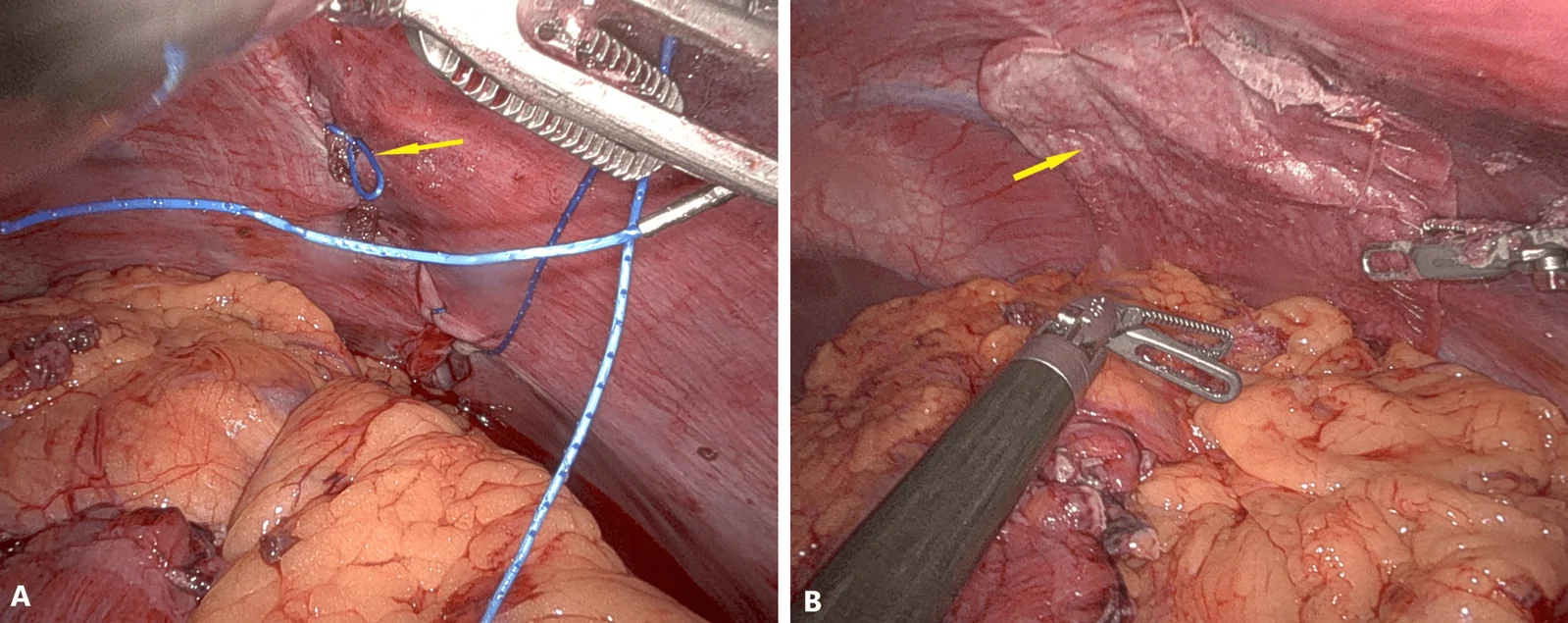
Robot-assisted repair. (A) The diaphragmatic defect was closed primarily using a running 2-0 nonabsorbable V-Loc™ suture (yellow arrow). (B) The primary repair was reinforced with a 10 × 10 cm XenMatrix™ Biologic Surgical Graft (Baxter).

The splenic flexure of the colon and omentum were incarcerated through a 7 × 4 cm defect in the left posterolateral diaphragm. Reduction required considerable traction because of chronic incarceration and dense adhesions (Figure 3). The herniated contents were reduced using atraumatic traction and careful adhesiolysis (Figure 4). During reduction, multiple superficial serosal tears occurred along the colon; however, the muscularis propria remained intact, and no full-thickness injury was identified. Given the superficial nature of these injuries, serosal repair was not performed. After reduction, the colon appeared viable based on its color, peristalsis, mesenteric pulsations, and fluorescence assessment. Therefore, bowel resection was not required. The diaphragmatic defect was closed primarily with a running 2-0 nonabsorbable V-Loc suture and reinforced with a 10 × 10 cm XenMatrix™ Biologic Surgical Graft (Baxter) (Figure 5), providing approximately 4 cm of overlap beyond the defect margins. Given the size of the defect, biologic mesh reinforcement was used to support the primary repair and reduce tension across the closure. The graft was secured with interrupted sutures and surgical adhesive. A chest tube was placed to manage the left pleural space. The estimated blood loss was approximately 250 mL, and the operative time was approximately two hours. No intraoperative complications occurred.

Postoperatively, the patient remained hemodynamically stable and maintained normal respiratory function without supplemental oxygen. The chest tube was removed after 24 hours without complications. Postoperative pain was adequately controlled, and his diet was gradually advanced without intolerance. Bowel function returned within 48 hours. The patient had an uncomplicated postoperative course and was discharged on postoperative day three. No postoperative complications or readmissions were reported during the three-month follow-up period.

## Discussion

A BH results from a congenital posterolateral diaphragmatic defect and is rarely diagnosed for the first time in adulthood. Recent reviews and case reports describe presentations ranging from incidental findings to acute gastrointestinal or respiratory emergencies, although the evidence base remains dominated by case reports and small case series [1-7,10]. Incidental fat-containing defects may be identified on cross-sectional imaging, whereas clinically significant hernias containing hollow viscera are much less common [1,2,4,5,10]. The condition is clinically important because it may cause obstruction, strangulation, ischemia, perforation, or cardiopulmonary compromise [1,3,6,10-12].

Symptoms depend on the herniated organ, defect size, and acuity of herniation. Gastrointestinal manifestations include abdominal or chest pain, nausea, vomiting, and obstruction, whereas respiratory manifestations include dyspnea, cough, and chest discomfort; some patients remain asymptomatic [1-7,10,16-18]. Our patient’s four-day course of abdominal pain, nausea, vomiting, and absence of bowel movements was consistent with an acute gastrointestinal presentation. CT is the imaging modality of choice because it can define the posterolateral defect, identify herniated organs and a transition point, and assess for complications [1-7,9-11,16,17]. Nevertheless, diagnostic errors can occur when intrathoracic bowel or fat is mistaken for pulmonary pathology or when the diaphragmatic defect is overlooked [2,5,7,18].

Operative repair is generally recommended for symptomatic adult BHs and is urgent when obstruction, incarceration, strangulation, perforation, volvulus, or respiratory compromise is suspected [1,3,8-14,17,18]. Management of truly asymptomatic incidental defects is less certain and should be tailored to each patient, considering the hernia contents, defect characteristics, operative risk, and patient preference [5,11,16]. In this case, a colonic transition point, inflammatory changes, persistent obstructive symptoms, and clinical dehydration justified urgent exploration to reduce the incarcerated contents and assess bowel viability.

Adult BHs have been repaired through laparotomy, thoracotomy, laparoscopy, thoracoscopy, combined minimally invasive approaches, and robot-assisted surgery [9-15,18]. A transabdominal route permits inspection of the abdominal viscera, reduction of the herniated bowel, and resection if ischemia is present, which is especially useful in cases of obstruction or incarceration. A transthoracic approach may facilitate the division of chronic intrathoracic adhesions and visualization of the thoracic side of the defect but provides less direct access for assessing the intra-abdominal bowel. Open surgery remains appropriate in cases of hemodynamic instability, perforation, extensive ischemia, inability to safely reduce the contents, or when minimally invasive repair is not feasible. Conventional laparoscopy offers smaller incisions and may support a shorter recovery than open surgery in selected patients, although suturing a posterior defect can be technically demanding [9,10,14]. Robotic systems add wristed instrumentation, stable, magnified 3D visualization, and intracorporeal suturing capability but also entail additional costs, setup time, platform availability, and the need for a trained team [15].

For this patient, the transabdominal robotic approach allowed direct evaluation of the obstructed colon after reduction and provided access to the posterolateral defect for primary closure and graft reinforcement. The robotic platform was selected because the patient was hemodynamically stable, the da Vinci Surgical System was available at our institution, and the procedure could be performed by a highly trained and experienced robotic surgical team, permitting a minimally invasive approach. The articulated instruments facilitated adhesiolysis, safe reduction of the herniated contents, posterior suturing, and graft fixation, while magnified 3D visualization assisted in accurately identifying anatomical structures and addressing technical challenges without intraoperative complications. Although this case demonstrates the feasibility of robotic repair, it does not establish that robotic surgery reduces complications, length of stay, or recurrence compared with conventional laparoscopy.

Published robotic experience remains sparse. Huang YJ and Fang YL described two robot-assisted adult CDH repairs, including one Bochdalek defect, with acceptable short-term outcomes [15]. That report and the present case support the feasibility of robot-assisted repair in selected adults, but differences in anatomy, symptoms, hernia contents, urgency, and repair technique preclude meaningful comparative conclusions. Other recent reports support laparoscopic, thoracoscopic, or combined minimally invasive repair in selected adults, yet they do not establish robotic superiority, and long-term follow-up remains inconsistent [9-14,17,18].

This report is limited by its single-patient design and the absence of a comparator. Selection bias and publication bias are substantial in a literature dominated by successful case reports. Multicenter registries or collaborative series with standardized reporting of defect size, hernia contents, bowel viability, operative approach, mesh use, conversion, complications, length of stay, patient-reported outcomes, and long-term recurrence would better clarify the role of robotic repair.

## Conclusions

BH in adults is an infrequent diagnosis and may present with nonspecific symptoms or acute complications, which can delay recognition and management. This case highlights that a robot-assisted approach can be used in the setting of an incarcerated diaphragmatic hernia, providing adequate exposure and facilitating precise dissection and repair. The enhanced articulation and visualization offered by the robotic platform may be advantageous in complex or confined operative fields. In our case, these features allowed effective reduction of the herniated contents and secure closure of the defect without major complications. While conventional minimally invasive techniques remain standard in many centers, robot-assisted surgery may be considered a reasonable alternative in selected patients when appropriate expertise and infrastructure are available. Additional studies are needed to clarify its comparative benefits and long-term outcomes in adult BH repair.
